# Post-traumatic symptoms after COVID-19 may (or may not) reflect disease severity

**DOI:** 10.1017/S003329172000481X

**Published:** 2020-11-27

**Authors:** James Badenoch, Benjamin Cross, Danish Hafeez, Jia Song, Cameron Watson, Matthew Butler, Timothy R. Nicholson, Alasdair G. Rooney, (The SARS-COV-neuro collaboration)

**Affiliations:** 1 Medical School, University of Birmingham, Birmingham, UK; 2 East Lancashire Hospitals NHS Trust, Lancashire, UK; 3 School of Medicine, The University of Manchester, Manchester, UK; 4 Deancross Personality Disorder Service, East London NHS Foundation Trust, London, UK; 5 Preventive Neurology Unit, Queen Mary University London, London, UK; 6 Institute of Psychiatry, Psychology and Neuroscience, King's College London, London, UK; 7 Centre for Clinical Brain Sciences, The University of Edinburgh, Edinburgh, UK

To the editor

We note emerging reports, including in this journal, of a high prevalence of post-traumatic stress disorder (PTSD) and post-traumatic stress (PTS) symptoms in patients with COVID-19 (Bo et al., 2020; Wesemann et al., 2020). These reports arise in the context of previous coronavirus pandemics in which PTSD occurred with a point-prevalence of 32.2% (Rogers et al., 2020). Although it may be presumed that this increased risk would translate across to COVID-19 patients, potential factors underlying this heightened vulnerability have received minimal attention.

It is well-recognised that severe critical illness can cause lasting psychological trauma. PTSD is an established sequela of intensive care admission, with pooled prevalence estimates of 17–44% after intensive care unit (ICU) care (Parker et al., 2015). The similarity of this prevalence range to that arising after SARS-CoV infection (Rogers et al., 2020) prompted the hypothesis that ICU admission could be a key driving factor behind the high prevalence of PTSD/PTS in patients with COVID-19. This hypothesis would predict that most patients with COVID-19 who develop PTSD/PTS were at one point critically ill and subsequently admitted to ICU. However, this has not been definitively established.

We are a research collaboration of clinical academics evaluating the neurology and neuropsychiatry of COVID-19. Our platform allows us to examine emerging trends in the COVID-19 neuropsychiatry literature (Butler et al., 2020). During that process and in partial contrast to the hypothesis that illness severity is the key driving factor in the development of PTSD, recent reports have emerged of PTS in patients with mild-moderate COVID-19. Estimates of the frequency of psychological trauma after mild-moderate disease currently vary. For example, Liu et al. (2020) evaluated PTS in 675 patients with predominantly mild-moderate COVID-19 disease, using the PTSD Checklist for DSM-5 (PCL-5). They found that 12.4% of patients had clinically meaningful PTS. By contrast, a far higher prevalence was reported by Bo et al. (2020) in this journal, who used the civilian version of a PTSD questionnaire based on DSM-IV (PCL-C). These authors found a prevalence of PTS of 96.2% in 714 patients with mild-moderate COVID-19 admitted to a government quarantine facility. How should we evaluate such a wide discrepancy?

Explanations may include methodological differences in how PTS symptoms were measured, the point within the course of COVID-19 when patients were assessed, and the setting in which assessment occurred. The studies by Liu et al. (2020) and Bo et al. (2020) used different and not directly comparable measures of PTS, and adopted different thresholds for ‘caseness’. The clinical populations were measured at different points in the course of COVID-19: Bo et al. (2020) assessed patients prior to discharge; the markedly higher prevalence in their study may have captured acute psychological distress during hospitalisation. Liu et al. (2020) assessed patients 1 month after discharge, capturing persistent symptoms only (and with arguably a more stringent definition of casesness). Finally, they were assessed in different settings. Government quarantine facilities (Bo et al., 2020) may provide only essential functions and might be a more distressing environment than being in a general hospital (Liu et al., 2020). These factors – case definition, point of assessment and place of treatment – presumably account for at least some of the heterogeneity in prevalence between the two studies. Nevertheless, they converge on the suggestion of frequent symptoms of PTS even in patients with mild-moderate COVID-19.

Other studies report high frequencies of PTS, but do not clearly describe COVID-19 severity. Wesemann et al. (2020) used the PCL-5 and found significant PTS in 8/19 (42%) COVID-19 patients shortly after their presentation to hospital, whereas Chang and Park (2020) used the PCL-5 to identify PTS in 13/64 (20%) patients assessed 10 weeks after discharge. Either study neither described COVID-19 severity nor indicated the requirement for critical care. Liu et al. (2020) reported both, but found that severity of COVID-19 was a significant risk factor for PTS (OR = 3.27), while ICU admission was not. This was surprising given the assumption that severity of COVID-19 and ICU admission correlates highly, and warrants further study.

Therefore at present, it is difficult to fully appraise the influence of COVID-19 severity to the development of PTSD. If potential factors underlying higher risk can be identified, it may be possible to identify patients vulnerable to developing persisting trauma, and intervene early. Several mechanisms have been advanced to explain how PTSD/PTS arises in patients with COVID-19. Biological and environmental factors may play a role, including in those with mild-moderate COVID-19. COVID-19 wards are characterised by patient isolation, use of extensive personal protective equipment by staff and lack of accessibility to relatives and visitors in hospital. Related to this alien environment, a contributory factor may be delirium. Several reports have demonstrated a high prevalence of delirium in COVID-19, not limited to patients in ICU but also affecting patients in general hospital wards with less severe disease (Mcloughlin et al., 2020). There is an emerging body of literature demonstrating that PTSD can be a complication of delirium (Bolton, Thilges, Lane, Lowe, & Mumby, 2019). Consequently, it would be interesting to ascertain whether delirium is associated with a greater likelihood of lasting psychological trauma in the specific context of COVID-19.

Taken together, the literature on PTSD/PTS in COVID-19 is still evolving. There is a natural tendency to assume that ICU admission and more severe disease will drive higher frequencies of lasting PTSD, and this may well be true, but we have noted emerging reports of a significant burden of psychological trauma in patients with less severe COVID-19. The importance of this phenomenon includes the opportunity it brings for early recognition and support, but also the implication for service planning: the number of mild-moderate COVID-19 patients will dwarf the number of patients requiring ICU, meaning that the former group may comprise the larger aggregate demand on healthcare and social resources. It is critical that we continue researching the complex milieu of biological and environmental factors which underlie heightened vulnerability to persisting PTS, including the potential mediating role of COVID-19 severity.
